# Supplementary material for: Exploring Captive Giant Panda Reproduction: Maternal and Offspring Factor Correlations from 324 Breeding Events
Source: Animals (Basel). 2025 Apr 21;15(8):1182. doi: 10.3390/ani15081182 (PMC12024069; doi:10.3390/ani15081182)
Supplement: Supplementary file 1 [file animals-15-01182-s001.zip › animals-3554820-supplementary.pdf]

Supplementary methods

1. General Approach

   All statistical analyses were performed using IBM SPSS Statistics (version 22.0) with a significance level of $\alpha = 0.05$. The analyses focused on evaluating relationships between maternal factors (maternal age, interbirth interval, gestational duration) and cub outcomes (cub birth weight, number of cubs per breeding events and neonatal mortality proportion). Below is a detailed breakdown of the methods applied for each hypothesis.

2. Variable Definitions and Data Handling

   - Continuous Variables:

     Cub birth weight, gestational duration: Assessed for normality using the Kolmogorov-Smirnov test.

   - Categorical Variables:

     Cub birth weight intervals (50-60, 60-70, 70-80, …, 220-230, 230-240, 240-250 g), interbirth intervals (0, 1, 2, 3, ≥4 years), gestational duration intervals (71–88, 89–110, 111–127, 128–150, 151–188 days): Expressed as percentages (proportions to the total breeding events).

3. Statistical Models by Hypothesis

   3.1 Maternal Age Effects

   - Twinning Rate (Figure 2A):

     Logistic regression with twin births proportions to the total breeding events as the response variable and maternal age as the predictor.

   - Cub Birth Weight (Figure 2B):

     One-way ANOVA with maternal age as fixed factors, cubs birth weight as response variable. Post-hoc pairwise comparisons used Bonferroni correction.

   - Neonatal Mortality (Figure 2C):

     Compared the neonatal mortality proportions to the total breeding events across maternal age.

   3.2 Gestational Duration Effects

   - Singleton vs. Twin (Figure 3A):

     Wilcoxon rank-sum test comparing number of cubs per breeding events and gestational duration.

   - Number of cubs per breeding events (Figure 3B):

     Compared the proportion of singleton vs. twin births across six gestational duration intervals (71–88, 89–110, 111–127, 128–150, 151–162, 165–188 days).

   - Cub birth weight (Figure 3C):

One-way ANOVA with gestational duration intervals as fixed factors, cubs birth weight as response variable. Post-hoc pairwise comparisons used Bonferroni correction.

- Neonatal Mortality (Figure 3D):
  Compared the neonatal mortality proportions across gestational duration intervals (6 groups).

3.3 Interbirth Interval Impacts

- Number of cubs per breeding events (Figure 4A):
  Compared proportions of singleton birth, twin birth and triplet births to total breeding events and interbirth interval categories.
- Cub birth weight (Figure 4B)
  One-way ANOVA with interbirth interval groups as fixed factors, cubs birth weight as responsible variables. Post-hoc pairwise comparisons used Bonferroni correction.
- Neonatal Mortality proportion (Figure 4C):
  Compared neonatal mortality proportions as the response and maternal age as predictors.

3.4 Cubs' outcomes

- Singleton vs. Multiple Birth Weight (Figure 5A):
  Wilcoxon rank-sum test comparing cub birth for singleton and multiple births (twin and triplet births).
- Twin Weight Differences and Mortality (Figure 5B):
  Wilcoxon rank-sum test comparing weight differences between twin pairs with vs. without mortality.

3.5 Temporal Trends in Birth Weight

One-way ANOVA across nine 3-year intervals (1998–2023), with Bonferroni-adjusted post-hoc tests (Figure 1C).

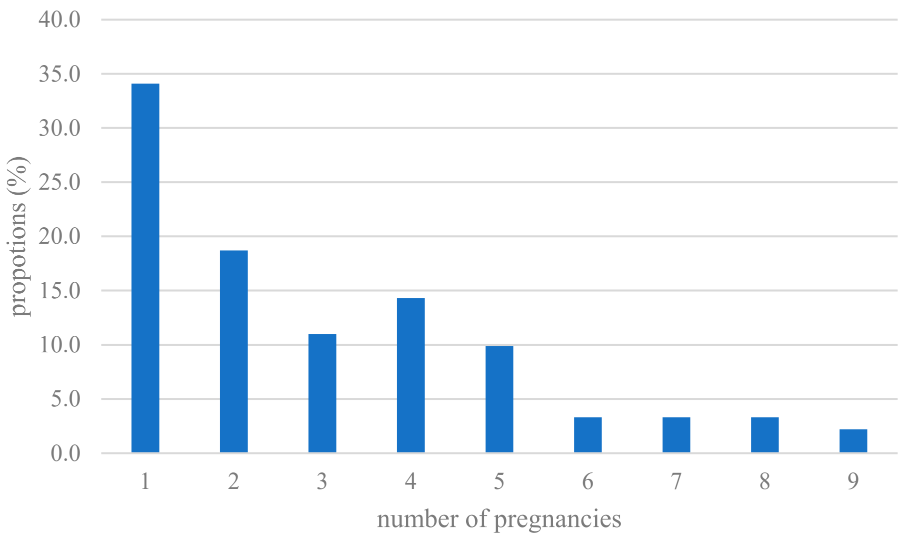

Figure S1. Distributions of number of pregnancies as percentages, n = 406 breeding events

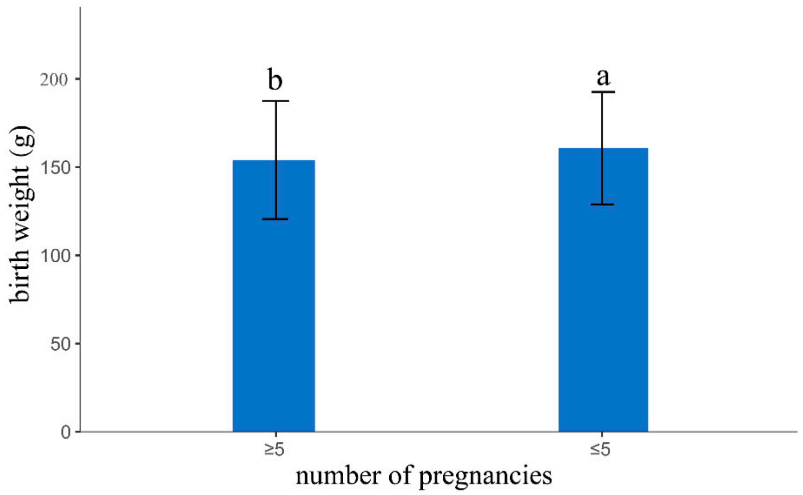

Figure S2. mean cub birth weight for number of pregnancies ⩾5 and ⩽5, n=406 births, error bars represent ± 1SD, different lowercase letters indicate differences according to ANOVA with post-hoc Bonferroni corrections, $P < 0.05$.

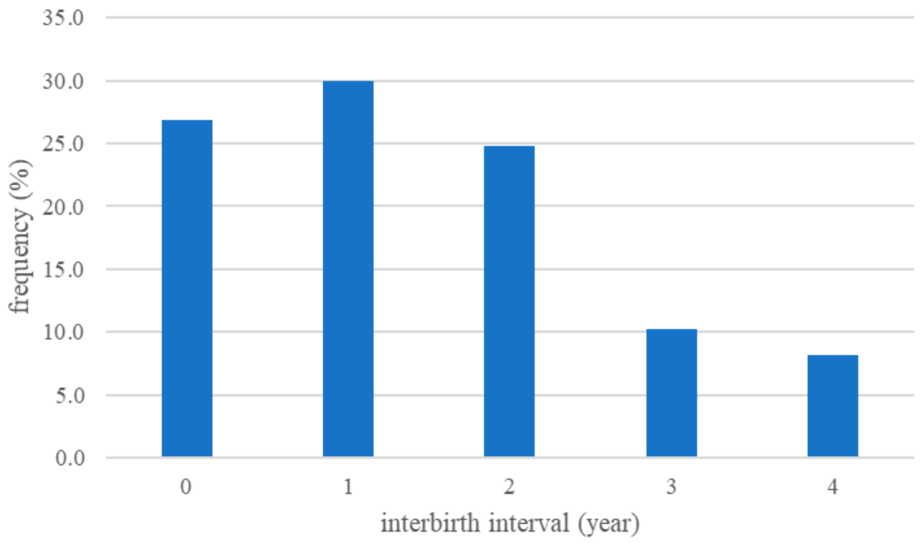

Figure S3. Distributions of interbirth intervals, n = 324 breeding events

Table S1. Maternal Age Distribution

Age (years)	Breeding Events	Percentage
5	16	5.0%
6	31	9.6%
7	32	9.9%
8	22	6.8%
9	25	7.7%
10	30	9.3%
11	39	12.0%
12	21	6.5%
13	21	6.5%
14	18	5.6%
15	15	4.6%
16	12	3.7%
17	12	3.7%
18	8	2.5%

Age (years)	Breeding Events	Percentage
19	7	2.2%
20	8	2.5%
21	2	0.6%
22	4	1.2%
23	1	0.3%

Table S2. Distributions of cub birth weight in 10g intervals (>a–≤b)

Weight Interval (g)	Number of Cubs	Percentage (%)
50-60	4	1.0
60-70	3	0.7
70-80	3	0.7
80-90	5	1.2
90-100	6	1.5
100-110	10	2.5
110-120	13	3.2
120-130	31	7.6
130-140	32	7.9
140-150	49	12.1
150-160	59	14.5
160-170	50	12.3
170-180	51	12.6
180-190	30	7.4
190-200	29	7.1
200-210	16	3.9
210-220	9	2.2
220-230	5	1.2
230-240	0	0

240-250	1	0.2

Table S3. Bonferroni Post-Hoc Multiple Comparisons of Mean Cub Birth Weight Between Time Period Groups (1998–2023) with Non-Significant Differences (All Adjusted P > 0.05, ANOVA)

Bonferroni's multiple comparisons test	Mean Diff.	95.00% CI of diff.	Adjusted P Value
2001-2003 vs.1998-2000	-5.67	-41.64 to 30.30	>0.9999
2004-2006 vs.1998-2000	5.54	-23.25 to 34.33	>0.9999
2007-2009 vs.1998-2000	0.1727	-27.63 to 27.98	>0.9999
2010-2012 vs.1998-2000	-1	-29.96 to 27.96	>0.9999
2013-2015 vs.1998-2000	-7.685	-33.97 to 18.60	>0.9999
2016-2018 vs.1998-2000	5.346	-19.55 to 30.24	>0.9999
2019-2021 vs.1998-2000	7.081	-18.43 to 32.59	>0.9999
2022-2023 vs.1998-2000	-0.4273	-29.57 to 28.71	>0.9999
2004-2006 vs.2001-2003	11.21	-22.20 to 44.62	>0.9999
2007-2009 vs.2001-2003	5.843	-26.72 to 38.41	>0.9999
2010-2012 vs.2001-2003	4.67	-28.89 to 38.23	>0.9999
2013-2015 vs.2001-2003	-2.015	-33.29 to 29.26	>0.9999
2016-2018 vs.2001-2003	11.02	-19.10 to 41.14	>0.9999
2019-2021 vs.2001-2003	12.75	-17.88 to 43.38	>0.9999
2022-2023 vs.2001-2003	5.243	-28.47 to 38.95	>0.9999
2007-2009 vs.2004-2006	-5.368	-29.77 to 19.04	>0.9999
2010-2012 vs.2004-2006	-6.54	-32.25 to 19.17	>0.9999
2013-2015 vs.2004-2006	-13.23	-35.88 to 9.428	>0.9999
2016-2018 vs.2004-2006	-0.194	-21.22 to 20.83	>0.9999
2019-2021 vs.2004-2006	1.54	-20.21 to 23.29	>0.9999
2022-2023 vs.2004-2006	-5.968	-31.88 to 19.94	>0.9999
2010-2012 vs.2007-2009	-1.173	-25.78 to 23.43	>0.9999
2013-2015 vs.2007-2009	-7.858	-29.25 to 13.53	>0.9999
2016-2018 vs.2007-2009	5.174	-14.49 to 24.83	>0.9999
2019-2021 vs.2007-2009	6.908	-13.53 to 27.34	>0.9999
2022-2023 vs.2007-2009	-0.6	-25.42 to 24.22	>0.9999
2013-2015 vs.2010-2012	-6.685	-29.55 to 16.18	>0.9999
2016-2018 vs.2010-2012	6.346	-14.91 to 27.60	>0.9999
2019-2021 vs.2010-2012	8.081	-13.90 to 30.06	>0.9999
2022-2023 vs.2010-2012	0.5727	-25.53 to 26.67	>0.9999
2016-2018 vs.2013-2015	13.03	-4.408 to 30.47	0.5971
2019-2021 vs.2013-2015	14.77	-3.542 to 33.07	0.3514
2022-2023 vs.2013-2015	7.258	-15.84 to 30.36	>0.9999
2019-2021 vs.2016-2018	1.734	-14.52 to 17.99	>0.9999
2022-2023 vs.2016-2018	-5.774	-27.28 to 15.73	>0.9999

2022-2023 vs.2019-2021	-7.508	-29.72 to 14.71	>0.9999

Bonferroni's multiple comparisons test was applied to control the family-wise error rate. Mean Diff. indicates the difference between group means. 95% CI of diff. represents the 95% confidence interval for the mean difference; intervals spanning zero imply non-significant differences. Adjusted P values account for multiple testing, with values >0.05 indicating no statistically significant differences (α=0.05). All comparisons in this analysis were non-significant.

Table S4. Bonferroni Post-Hoc Multiple Comparisons of Mean Cub Birth Weight Between Maternal Age (5–23) with Non-Significant Differences (All Adjusted P > 0.05, ANOVA)

Bonferroni's multiple comparisons test	Mean Diff.	95.00% CI of diff.	Adjusted P Value
6 vs. 5	0.7361	-41.31 to 42.78	>0.9999
7 vs. 5	5.419	-35.60 to 46.44	>0.9999
8 vs. 5	8.139	-32.40 to 48.68	>0.9999
9 vs. 5	12.25	-29.14 to 53.64	>0.9999
10 vs. 5	11.01	-29.84 to 51.86	>0.9999
11 vs. 5	7.963	-31.15 to 47.07	>0.9999
12 vs. 5	0.1406	-40.88 to 41.16	>0.9999
13 vs. 5	13.85	-30.00 to 57.70	>0.9999
14 vs. 5	-2.092	-45.24 to 41.06	>0.9999
15 vs. 5	12.58	-35.09 to 60.25	>0.9999
16 vs. 5	-6.142	-53.07 to 40.79	>0.9999
17 vs. 5	-7.925	-55.60 to 39.75	>0.9999
18 vs. 5	2.675	-52.63 to 57.98	>0.9999
19 vs. 5	-15.15	-70.46 to 40.16	>0.9999
20 vs. 5	-25.32	-78.75 to 28.11	>0.9999
21 vs. 5	-5.075	-97.62 to 87.47	>0.9999
22 vs. 5	-4.975	-74.94 to 64.99	>0.9999
23 vs. 5	-4.125	-96.67 to 88.42	>0.9999
7 vs. 6	4.683	-26.98 to 36.35	>0.9999
8 vs. 6	7.403	-23.63 to 38.44	>0.9999
9 vs. 6	11.51	-20.63 to 43.66	>0.9999
10 vs. 6	10.28	-21.17 to 41.72	>0.9999
11 vs. 6	7.226	-21.92 to 36.38	>0.9999
12 vs. 6	-0.5955	-32.26 to 31.07	>0.9999
13 vs. 6	13.12	-22.14 to 48.37	>0.9999
14 vs. 6	-2.829	-37.21 to 31.56	>0.9999
15 vs. 6	11.85	-28.06 to 51.75	>0.9999
16 vs. 6	-6.878	-45.90 to 32.14	>0.9999
17 vs. 6	-8.661	-48.57 to 31.25	>0.9999

18 vs. 6	1.939	-46.84 to 50.72	>0.9999
19 vs. 6	-15.89	-64.66 to 32.89	>0.9999
20 vs. 6	-26.06	-72.70 to 20.58	>0.9999
21 vs. 6	-5.811	-94.61 to 82.99	>0.9999
22 vs. 6	-5.711	-70.63 to 59.21	>0.9999
23 vs. 6	-4.861	-93.66 to 83.94	>0.9999
8 vs. 7	2.721	-26.92 to 32.36	>0.9999
9 vs. 7	6.83	-23.97 to 37.62	>0.9999
10 vs. 7	5.594	-24.47 to 35.66	>0.9999
11 vs. 7	2.544	-25.11 to 30.20	>0.9999
12 vs. 7	-5.278	-35.57 to 25.02	>0.9999
13 vs. 7	8.435	-25.60 to 42.47	>0.9999
14 vs. 7	-7.511	-40.64 to 25.61	>0.9999
15 vs. 7	7.163	-31.67 to 45.99	>0.9999
16 vs. 7	-11.56	-49.48 to 26.36	>0.9999
17 vs. 7	-13.34	-52.17 to 25.49	>0.9999
18 vs. 7	-2.744	-50.64 to 45.16	>0.9999
19 vs. 7	-20.57	-68.47 to 27.33	>0.9999
20 vs. 7	-30.74	-76.46 to 14.98	>0.9999
21 vs. 7	-10.49	-98.81 to 77.83	>0.9999
22 vs. 7	-10.39	-74.66 to 53.87	>0.9999
23 vs. 7	-9.544	-97.86 to 78.78	>0.9999
9 vs. 8	4.109	-26.04 to 34.26	>0.9999
10 vs. 8	2.874	-26.53 to 32.28	>0.9999
11 vs. 8	-0.1768	-27.11 to 26.76	>0.9999
12 vs. 8	-7.999	-37.64 to 21.64	>0.9999
13 vs. 8	5.714	-27.73 to 39.16	>0.9999
14 vs. 8	-10.23	-42.76 to 22.29	>0.9999
15 vs. 8	4.443	-33.88 to 42.76	>0.9999
16 vs. 8	-14.28	-51.68 to 23.11	>0.9999
17 vs. 8	-16.06	-54.38 to 22.25	>0.9999
18 vs. 8	-5.464	-52.95 to 42.02	>0.9999
19 vs. 8	-23.29	-70.78 to 24.20	>0.9999
20 vs. 8	-33.46	-78.75 to 11.83	>0.9999
21 vs. 8	-13.21	-101.3 to 74.88	>0.9999
22 vs. 8	-13.11	-77.07 to 50.84	>0.9999
23 vs. 8	-12.26	-100.4 to 75.83	>0.9999
10 vs. 9	-1.235	-31.80 to 29.33	>0.9999
11 vs. 9	-4.286	-32.49 to 23.92	>0.9999
12 vs. 9	-12.11	-42.90 to 18.69	>0.9999
13 vs. 9	1.605	-32.87 to 36.08	>0.9999
14 vs. 9	-14.34	-47.92 to 19.24	>0.9999
15 vs. 9	0.3338	-38.89 to 39.55	>0.9999
16 vs. 9	-18.39	-56.71 to 19.93	>0.9999

17 vs. 9	-20.17	-59.39 to 19.05	>0.9999
18 vs. 9	-9.573	-57.79 to 38.64	>0.9999
19 vs. 9	-27.4	-75.62 to 20.82	>0.9999
20 vs. 9	-37.57	-83.62 to 8.486	0.5196
21 vs. 9	-17.32	-105.8 to 71.17	>0.9999
22 vs. 9	-17.22	-81.72 to 47.28	>0.9999
23 vs. 9	-16.37	-104.9 to 72.12	>0.9999
11 vs. 10	-3.05	-30.45 to 24.35	>0.9999
12 vs. 10	-10.87	-40.94 to 19.19	>0.9999
13 vs. 10	2.841	-30.98 to 36.67	>0.9999
14 vs. 10	-13.11	-46.02 to 19.81	>0.9999
15 vs. 10	1.569	-37.08 to 40.22	>0.9999
16 vs. 10	-17.15	-54.89 to 20.58	>0.9999
17 vs. 10	-18.94	-57.59 to 19.71	>0.9999
18 vs. 10	-8.338	-56.09 to 39.42	>0.9999
19 vs. 10	-26.16	-73.92 to 21.59	>0.9999
20 vs. 10	-36.33	-81.90 to 9.236	0.6429
21 vs. 10	-16.09	-104.3 to 72.15	>0.9999
22 vs. 10	-15.99	-80.14 to 48.17	>0.9999
23 vs. 10	-15.14	-103.4 to 73.10	>0.9999
12 vs. 11	-7.822	-35.48 to 19.83	>0.9999
13 vs. 11	5.891	-25.81 to 37.59	>0.9999
14 vs. 11	-10.05	-40.78 to 20.67	>0.9999
15 vs. 11	4.62	-32.19 to 41.43	>0.9999
16 vs. 11	-14.1	-49.95 to 21.74	>0.9999
17 vs. 11	-15.89	-52.69 to 20.92	>0.9999
18 vs. 11	-5.287	-51.56 to 40.99	>0.9999
19 vs. 11	-23.11	-69.39 to 23.16	>0.9999
20 vs. 11	-33.28	-77.30 to 10.73	>0.9999
21 vs. 11	-13.04	-100.5 to 74.41	>0.9999
22 vs. 11	-12.94	-76.00 to 50.12	>0.9999
23 vs. 11	-12.09	-99.54 to 75.36	>0.9999
13 vs. 12	13.71	-20.32 to 47.74	>0.9999
14 vs. 12	-2.233	-35.36 to 30.89	>0.9999
15 vs. 12	12.44	-26.39 to 51.27	>0.9999
16 vs. 12	-6.282	-44.20 to 31.64	>0.9999
17 vs. 12	-8.066	-46.89 to 30.76	>0.9999
18 vs. 12	2.534	-45.36 to 50.43	>0.9999
19 vs. 12	-15.29	-63.19 to 32.61	>0.9999
20 vs. 12	-25.46	-71.18 to 20.26	>0.9999
21 vs. 12	-5.216	-93.54 to 83.11	>0.9999
22 vs. 12	-5.116	-69.38 to 59.15	>0.9999
23 vs. 12	-4.266	-92.59 to 84.06	>0.9999
14 vs. 13	-15.95	-52.52 to 20.63	>0.9999

15 vs. 13	-1.271	-43.08 to 40.54	>0.9999
16 vs. 13	-20	-60.96 to 20.97	>0.9999
17 vs. 13	-21.78	-63.59 to 20.03	>0.9999
18 vs. 13	-11.18	-61.52 to 39.17	>0.9999
19 vs. 13	-29	-79.35 to 21.34	>0.9999
20 vs. 13	-39.17	-87.45 to 9.104	0.5465
21 vs. 13	-18.93	-108.6 to 70.74	>0.9999
22 vs. 13	-18.83	-84.94 to 47.28	>0.9999
23 vs. 13	-17.98	-107.6 to 71.69	>0.9999
15 vs. 14	14.67	-26.40 to 55.75	>0.9999
16 vs. 14	-4.049	-44.27 to 36.17	>0.9999
17 vs. 14	-5.833	-46.91 to 35.24	>0.9999
18 vs. 14	4.767	-44.97 to 54.51	>0.9999
19 vs. 14	-13.06	-62.80 to 36.68	>0.9999
20 vs. 14	-23.23	-70.87 to 24.42	>0.9999
21 vs. 14	-2.983	-92.31 to 86.35	>0.9999
22 vs. 14	-2.883	-68.53 to 62.76	>0.9999
23 vs. 14	-2.033	-91.36 to 87.30	>0.9999
16 vs. 15	-18.72	-63.75 to 26.31	>0.9999
17 vs. 15	-20.51	-66.31 to 25.29	>0.9999
18 vs. 15	-9.907	-63.61 to 43.80	>0.9999
19 vs. 15	-27.73	-81.44 to 25.97	>0.9999
20 vs. 15	-37.9	-89.67 to 13.87	>0.9999
21 vs. 15	-17.66	-109.3 to 73.94	>0.9999
22 vs. 15	-17.56	-86.26 to 51.14	>0.9999
23 vs. 15	-16.71	-108.3 to 74.89	>0.9999
17 vs. 16	-1.783	-46.81 to 43.25	>0.9999
18 vs. 16	8.817	-44.23 to 61.87	>0.9999
19 vs. 16	-9.008	-62.06 to 44.04	>0.9999
20 vs. 16	-19.18	-70.27 to 31.91	>0.9999
21 vs. 16	1.067	-90.15 to 92.28	>0.9999
22 vs. 16	1.167	-67.02 to 69.36	>0.9999
23 vs. 16	2.017	-89.20 to 93.23	>0.9999
18 vs. 17	10.6	-43.11 to 64.31	>0.9999
19 vs. 17	-7.225	-60.93 to 46.48	>0.9999
20 vs. 17	-17.39	-69.17 to 34.38	>0.9999
21 vs. 17	2.85	-88.75 to 94.45	>0.9999
22 vs. 17	2.95	-65.75 to 71.65	>0.9999
23 vs. 17	3.8	-87.80 to 95.40	>0.9999
19 vs. 18	-17.83	-78.41 to 42.76	>0.9999
20 vs. 18	-27.99	-86.88 to 30.89	>0.9999
21 vs. 18	-7.75	-103.5 to 88.05	>0.9999
22 vs. 18	-7.65	-81.85 to 66.55	>0.9999
23 vs. 18	-6.8	-102.6 to 89.00	>0.9999

20 vs. 19	-10.17	-69.05 to 48.71	>0.9999
21 vs. 19	10.08	-85.72 to 105.9	>0.9999
22 vs. 19	10.18	-64.03 to 84.38	>0.9999
23 vs. 19	11.03	-84.77 to 106.8	>0.9999
21 vs. 20	20.24	-74.48 to 115.0	>0.9999
22 vs. 20	20.34	-52.47 to 93.16	>0.9999
23 vs. 20	21.19	-73.53 to 115.9	>0.9999
22 vs. 21	0.1	-104.8 to 105.0	>0.9999
23 vs. 21	0.95	-120.2 to 122.1	>0.9999
23 vs. 22	0.85	-104.1 to 105.8	>0.9999

Bonferroni's multiple comparisons test was applied to control the family-wise error rate. Mean Diff. indicates the difference between group means. 95% CI of diff. represents the 95% confidence interval for the mean difference; intervals spanning zero imply non-significant differences. Adjusted P values account for multiple testing, with values >0.05 indicating no statistically significant differences (α=0.05). All comparisons in this analysis were non-significant.

Table S5. Bonferroni Post-Hoc Multiple Comparisons of Mean Cub Birth Weight Between 6 Grouped Gestational Durations with Non-Significant Differences (All Adjusted P > 0.05, ANOVA)

Bonferroni's multiple comparisons test	Mean Diff.	95.00% CI of diff.	Adjusted P Value
94-109d vs. 71-88d	2.835	-39.31 to 44.98	>0.9999
110-127d vs. 71-88d	-1.955	-43.24 to 39.33	>0.9999
128-145d vs. 71-88d	4.146	-37.23 to 45.52	>0.9999
146-161d vs. 71-88d	-4.175	-47.97 to 39.62	>0.9999
165-188d vs. 71-88d	2.842	-50.57 to 56.25	>0.9999
110-127d vs. 94-109d	-4.791	-19.63 to 10.04	>0.9999
128-145d vs. 94-109d	1.311	-13.77 to 16.39	>0.9999
146-161d vs. 94-109d	-7.01	-27.83 to 13.81	>0.9999
165-188d vs. 94-109d	0.006343	-36.99 to 37.00	>0.9999
128-145d vs. 110-127d	6.101	-6.373 to 18.58	>0.9999
146-161d vs. 110-127d	-2.219	-21.24 to 16.80	>0.9999
165-188d vs. 110-127d	4.797	-31.21 to 40.81	>0.9999
146-161d vs. 128-145d	-8.32	-27.53 to 10.89	>0.9999
165-188d vs. 128-145d	-1.304	-37.42 to 34.81	>0.9999
165-188d vs. 146-161d	7.016	-31.85 to 45.88	>0.9999

Bonferroni's multiple comparisons test was applied to control the family-wise error rate. Mean Diff. indicates the difference between group means. 95% CI of diff. represents the 95% confidence interval for the mean difference; intervals spanning zero imply non-significant differences. Adjusted P values account for multiple testing, with values >0.05 indicating no statistically significant differences (α=0.05). All comparisons in this analysis were non-significant.

Table S6. Bonferroni Post-Hoc Multiple Comparisons of Mean Cub Birth Weight Between Interbirth Intervals with Non-Significant Differences (All Adjusted P > 0.05, ANOVA)

Bonferroni's multiple comparisons test	Mean Diff.	95.00% CI of diff.	Adjusted P Value
1 vs. 0	4.382	-8.641 to 17.41	>0.9999
2 vs. 0	3.875	-9.653 to 17.40	>0.9999
3 vs. 0	0.7727	-17.37 to 18.91	>0.9999
≥4 vs. 0	-11.94	-31.09 to 7.207	0.7919
2 vs. 1	-0.5069	-13.65 to 12.64	>0.9999
3 vs. 1	-3.609	-21.46 to 14.25	>0.9999
≥4 vs. 1	-16.33	-35.21 to 2.557	0.1511
3 vs. 2	-3.102	-21.33 to 15.12	>0.9999
≥4 vs. 2	-15.82	-35.05 to 3.415	0.2078
≥4 vs. 3	-12.72	-35.43 to 9.998	>0.9999

4 represents an interbirth interval of 4 years or more

Bonferroni's multiple comparisons test was applied to control the family-wise error rate. Mean Diff. indicates the difference between group means. 95% CI of diff. represents the 95% confidence interval for the mean difference; intervals spanning zero imply non-significant differences. Adjusted P values account for multiple testing, with values >0.05 indicating no statistically significant differences (α=0.05). All comparisons in this analysis were non-significant.
